# Restorative materials without Bis-GMA – myth or reality?

**DOI:** 10.1080/07853890.2021.1897387

**Published:** 2021-09-28

**Authors:** Hermano Garcia, Inês Caldeira Fernandes, Jorge Caldeira, Alexandra Pinto, Inês Carpinteiro, Ana Azul

**Affiliations:** a Instituto Universitário Egas Moniz (IUEM), Egas Moniz Cooperativa de Ensino Superior, Caparica, Portugal; b Centro de Investigação Interdisciplinar Egas Moniz (CiiEM), Egas Moniz Cooperativa de Ensino Superior, Caparica, Portugal

## Abstract

**Introduction:**

The Bis-GMA, which is known to possess toxic properties to the human body, is part of the chemical structure of several direct dental restorative materials [1]. However, currently, there are composite resins on the market that are free of this monomer [2]. From the dental resin are released many other compounds [3]. The aim of this study was to analyse the composition of restorative materials from three different brands, and to verify whether or not Bis-GMA monomers are present in their composition.

**Materials and methods:**

Samples of the Enamel *plus* HRi® Universal Dentine (Micerium), Enamel *plus* HRi^®^ Bio Function (Micerium), Filtek^®^ One Bulk Fill Restorative (3 M ESPE) and Admira^®^ Fusion (VOCO) resins were prepared using three different methods: light-curing resin specimens placed for 18 min in an ethanol/water solution; light-curing resin specimens placed for 180 min in an ethanol/water solution; resin samples eluted in an acetonitrile solution without being photopolymerized. These samples were analysed using the HPLC (High Performance Liquid Chromatography) technique.

**Results:**

HEMA, Bis-GMA and UDMA monomers were found in the composite resin Enamel *plus* HRi^®^ Universal Dentine (Micerium); UDMA monomers were detected in Enamel *plus* HRi^®^ Bio Function (Micerium); UDMA and TEGDMA monomers were revealed in Filtek^®^ One Bulk Fill Restorative (3 M ESPE), and HEMA and UDMA peaks were obtained in Admira^®^ Fusion (VOCO).

**Discussion and conclusions:**

The release of most of the residual monomers occurs in the first minutes after polymerisation. As shown in the results, the difference of the concentrations of monomers, detected by the HPLC method, between the resins eluted during 18 min and 180 min, was not significant. It was concluded that in samples of non-polymerized resins, it is possible to detect a larger number of residual monomers, and that Bis-GMA is only present in Enamel *plus* HRi® Universal Dentine (Micerium) resin.
